# Protein cleavage strategies for an improved analysis of the membrane proteome

**DOI:** 10.1186/1477-5956-4-2

**Published:** 2006-03-02

**Authors:** Frank Fischer, Ansgar Poetsch

**Affiliations:** 1 Plant Biochemistry, Ruhr University Bochum, Universitaetsstrasse 150, Bochum, Germany

## Abstract

**Background:**

Membrane proteins still remain elusive in proteomic studies. This is in part due to the distribution of the amino acids lysine and arginine, which are less frequent in integral membrane proteins and almost absent in transmembrane helices. As these amino acids are cleavage targets for the commonly used protease trypsin, alternative cleavage conditions, which should improve membrane protein analysis, were tested by *in silico *digestion for the three organisms *Saccharomyces cerevisiae, Halobacterium sp. NRC-1*, and *Corynebacterium glutamicum *as hallmarks for eukaryotes, archea and eubacteria.

**Results:**

For the membrane proteomes from all three analyzed organisms, we identified cleavage conditions that achieve better sequence and proteome coverage than trypsin. Greater improvement was obtained for bacteria than for yeast, which was attributed to differences in protein size and GRAVY. It was demonstrated for bacteriorhodopsin that the *in silico *predictions agree well with the experimental observations.

**Conclusion:**

For all three examined organisms, it was found that a combination of chymotrypsin and staphylococcal peptidase I gave significantly better results than trypsin. As some of the improved cleavage conditions are not more elaborate than trypsin digestion and have been proven useful in practice, we suppose that the cleavage at both hydrophilic and hydrophobic amino acids should facilitate in general the analysis of membrane proteins for all organisms.

## Background

Major achievements have been accomplished in the field of mass spectrometry, namely the mild ionization techniques of MALDI [1] and ESI [2], which have been honoured in 2002 by the Nobel Prize in chemistry [3]. These breakthroughs have raised protein analysis to a level, where in one experiment the study of the complete cellular set of all proteins, the proteome, has become possible. For the task of protein identification, nowadays MALDI and ESI ionisation sources are employed that are commonly coupled to quadrupols, TOFs or ion traps for ion separation. The proteins are cleaved with chemicals or proteases to obtain smaller peptide fragments. In usual high-throughput MALDI experiments, peptides are measured in the range of 600–4000 Da. For ESI, either a quadrupole with a common mass range of 0 to ~4000 Da or an ion trap with a common mass range of ~600 to ~4000 Da is used for molecule separation and analysis. The layout of ESI instruments is highly suitable for molecule fragmentation by collision with an inert gas, which can be used to determine the amino acid sequence of the peptide from the mass differences of the fragments. Nonetheless, the technology to study the proteome, proteomics, is far from mature. Certain protein classes, especially integral membrane proteins, still remain elusive. Different, complementary separation methods exist for integral membrane proteins. One possibility is to separate solubilized, intact membrane proteins, which can be carried out with blue native electrophoresis, SDS-PAGE, chromatography, and combinations of the techniques [4]. Alternatively, the membrane fraction is treated with proteases to yield peptide fragments that are separated by multidimensional chromatography, e.g. MudPIT [5] and their sequences determined automatically, for instance with the SEQUEST algorithm [6]. Several factors may hamper the detection of integral membrane proteins, such as solubilization problems, and low protein abundance. Another problem is the inability to obtain peptides from protein cleavage that are suitable for protein identification. The amino acids lysine and arginine are less frequent in membrane proteins compared to cytosolic proteins [7]. Furthermore, these positively charged amino acids are not uniformly distributed along the protein sequence, but are mainly present in hydrophilic domains and almost absent in transmembrane helices. Hence the commonly used protease trypsin is unfavorable for the cleavage of integral membrane proteins. Alternative cleavage conditions, such as the consecutive use of cyanogen bromide and trypsin [8], as well as other proteases like proteinase K have been described [9] and proven superior to trypsin in practice. However, the latter is only suitable if MS/MS technologies can be employed for protein identification and it generally produces complex peptide mixtures. Furthermore, no transmembrane (TM) domains were detected by proteinase K treatment due to experimental limitations. As an efficient solubilization of membrane proteins is a necessary prerequisite for their identification by mass spectrometry and the detection of TM domains, new detergents [10] or solubilization conditions employing organic solvents [11] were developed. Yet far more possible cleavage conditions exist than experimentally tested for membrane proteins, but it is often not feasible to test them all in practice. A faster alternative for the screening for new, superior cleavage conditions is to perform *in silico *digestion (i.e. computational simulation) of entire membrane proteomes. *In silico *digestion has been carried out before to determine the suitability of single cleavage reagents [12], and to perform statistical analysis of proteomes [13]. However, it has not been performed on the whole proteome scale to discover better cleavage conditions for integral membrane proteins, nor have the membrane proteomes from eukaryotes, archae, and prokaryotes been compared.

Taking the mass range of common instruments, and the MS/MS capability of ESI into account, *in silico *proteome analysis was carried out to determine the best experimental procedures for the identification of integral membrane proteins from *Saccharomyces cerevisiae, Halobacterium sp. NRC-1*, and *Corynebacterium glutamicum*. We show that the physiochemical properties of the membrane proteins significantly differ between the three kinds of organisms with consequences for protein identification by mass spectrometry. Finally, the practical applicability of new and potentially superior cleavage conditions were tested for bacteriorhodopsin as model membrane protein.

## Results

### Physicochemical features of the membrane proteomes

**Figure 1:**
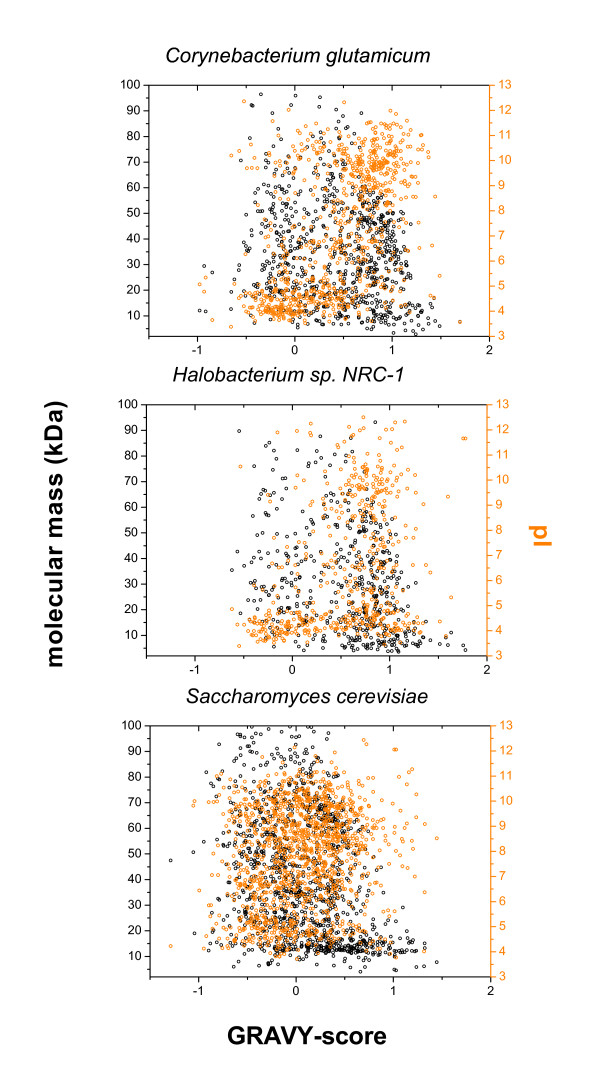
**Plot of molecular weight and pI versus GRAVY-score**. The pI and GRAVY-scores are plotted for the membrane proteomes of *C. glutamicum, Halobacterium sp., and S. cerevisiae*. GRAVY-scores were calculated according to the values from Kyte and Doolittle [7].

The GRAVY (Grand Average of HydropathY), molecular weight and pI were calculated for *S. cerevisiae, Halobacterium sp*., and *C. glutamicum *and are depicted in Fig. 1. According to the TMHMM algorithm, the organisms possess the following number of integral membrane proteins: 1264 (27% of all proteins) for *S. cerevisiae*, 508 (21% of all) for *Halobacterium sp*., and 639 (21% of all) for *C. glutamicum*. It is evident for all three organisms that the small proteins (below 20 kDa) are most hydrophobic. The trend towards a smaller GRAVY-score with increasing protein size is observed for all organisms: Membrane proteins have an average GRAVY-score of 0.32 (< 20 kDa) and -0.15 (>70 kDa) in *S. cerevisiae*, an average GRAVY-score of 0.70 (<20 kDa) and 0.17 (>70 kDa) in *Halobacterium sp*., and an average GRAVY-score of 0.53 (< 20 kDa) and 0.15 (> 70 kDa) in *C. glutamicum*. In comparison to *S. cerevisae*, the GRAVY-score distribution is shifted to higher values for the prokaryote *C. glutamicum *and the archae *Halobacterium sp*., thus the membrane proteins of the latter two are on average more hydrophobic than in yeast. This is also reflected in the mean GRAVY-score, which is 0.06 for *S. cerevisiae*, 0.43 for *C. glutamicum*, and 0.54 for *Halobacterium sp*. The pI distribution of proteins in yeast is trimodal, while it is bimodal for the other two organisms. In *C. glutamicum *most of the acidic membrane proteins are more hydrophilic, while the basic ones are more hydrophobic. In *Halobacterium sp*. about half of the acidic membrane proteins are hydrophilic, while most of the basic membrane proteins are hydrophobic. In yeast, there is also a trend from hydrophilic to hydrophobic with increasing pI, but it is less distinct than in the other two organisms. The mean pI of the membrane proteome is 7.8 for yeast, 7.3 for *C. glutamicum*, and 6.5 for the halobacterium. The mean size of the integral membrane proteins increases from 31 kDa for *Halobacterium sp*., 36 kDa for *C. glutamicum *to 49 kDa for yeast. In summary, this data suggest that membrane proteomics will be more difficult for prokaryotes and archea as for yeast, since the membrane proteins of the former two groups are on average more hydrophobic and smaller.

### Protein sequence coverage of peptide fragments

**Figure 2:**
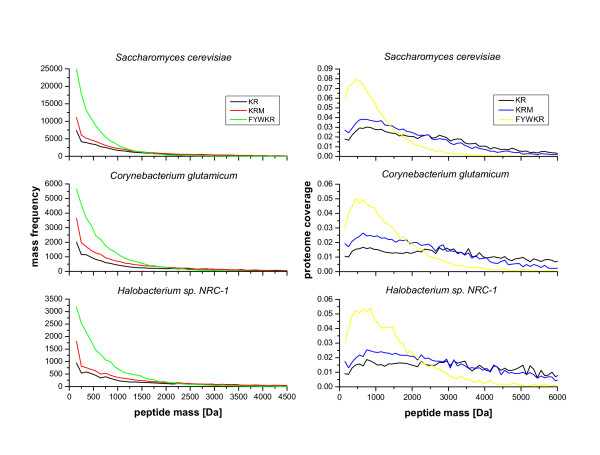
**Dependence of membrane proteome coverage and peptide frequency on peptide mass**. For the cleavages with trypsin (KR), trypsin/cyanogen bromide (KRM), and trypsin/chymotrypsin (FYWKR) the frequency of peptide masses is depicted in the range from 0–4500 Da (Fig. 2A). For better clarity, cleavages that result in the release of a single amino acid were not counted. With these values, a length normalized value was calculated to obtain the membrane proteome coverage in relation to the peptide mass (Fig. 2B). The displayed value is per mass coverage of the total membrane proteome for the respective organism.

High sequence coverage is desirable for the analysis of posttranslational protein modifications (e.g. phosphorylation, glycosylation). Furthermore, it can be an indication how well suited a digestion condition is for protein identification by MALDI-TOF PMF (peptide mass fingerprinting). For the latter, the total number of peptides in the mass window should be maximized, which often correlates with the highest sequence coverage. Protein sequence coverages were calculated based on their corresponding peptides for different *in silico *digestion conditions. For three selected cleavage conditions, i.e. trypsin (KR), trypsin + cyanogen bromide (KRM), and trypsin + chymotrypsin (FYWKR) the frequency of peptides with different masses is plotted in Fig. 2A. Cleavage sites are given in one letter amino acid code in the figures; the different digestion conditions are simulations for single proteases / chemicals, or combinations of them (see Tab. 1). For all three organisms, the number of obtained peptides decreases exponentially with peptide mass. Furthermore, cleavage at more amino acid residues leads to a higher number of peptides mainly below a mass of 2000 Da. Although the number of peptides above 2000 Da is rather small, their contribution to the total proteome coverage must not be neglected. Therefore, the proteome coverage in relation to the number of peptides with a certain mass was calculated for all three organisms (Fig. 2B). It can be seen for *S. cerevisae*and trypsin cleavage that peptides with ~750 Da mass contribute 3% to the total membrane proteome coverage and much smaller number of peptides with ~4000 Da still contribute 1%. Cleavage at more residues enlarges the difference in coverage between smaller and larger peptides. Use of trypsin and cyanogen bromide results in membrane proteome coverage of 3.8% with peptides ~750 Da in mass and coverage of 0.7% with peptides ~4000 Da in mass. Cleavage with the combination of trypsin and chymotrypsin yields coverage of 6.7% with ~750 Da peptides and 0.01% with ~4000 Da peptides. In the diagram for *C. glutamicum*, it can be seen that peptides with ~750 Da mass contribute 1.6% to the total membrane proteome coverage and peptides with ~4000 Da contribute almost 1% after digestion with trypsin. Use of trypsin and cyanogen bromide results in membrane proteome coverage of 2.5% with peptides ~750 Da in mass and coverage of 0.9% with peptides ~4000 Da in mass. Cleavage with the combination of trypsin and chymotrypsin yields coverage of 4.6% with ~750 Da peptides and 0.017% with ~4000 Da peptides. For *Halobacterium sp*. and trypsin cleavage the membrane coverage is almost equal for small and large peptides: 1.9% for ~750 Da peptides and 1.1% for ~4000 Da peptides. A slightly larger difference in membrane proteome coverage can be observed if trypsin and cyanogen bromide are used for cleavage: 2.5% for ~750 Da peptides and 1.1% for ~4000 Da peptides. A combined cleavage with trypsin and chymotrypsin results in membrane proteome coverage of 5.3% with peptides ~750 Da in mass and coverage of 0.03% with peptides ~4000 Da in mass.

**Table 1:** Abbreviations used for the cleavage conditions

KR	Trypsin	KRM	Trypsin/cyanogen bromide
FYW	Chymotrypsin (specific)	FYWL	Chymotrypsin (unspecific)
KRW	Trypsin/ iodosobenzoic acid	FYWKR	Trypsin / chymotrypsin
FYWLM	Chymotrypsin/ cyanogen bromide	FYWE	Chymotrypsin / staphylococcal peptidase I
KRMW	Trypsin / iodosobenzoic acid / cyanogen bromide	FYWK	Chymotrypsin / endopeptidase Lys-C
KRDE	Trypsin / staphylococcal peptiase I / endopeptidase Asp-N	KRE	Trypsin / staphylococcal peptiase I
MST	Pentafluoropropionic acid / cyanogen bromide		

In a more comprehensive analysis a variety of cleavage conditions was tested and the percentage of peptides in the mass detection window of 600–4000 Da was calculated. This mass window is typical for common MALDI-TOF instruments and ESI ion traps, though for some peptides and instruments the window may be larger or smaller in reality.

In the first step, analysis of the complete *C. glutamicum *proteome (integral membrane and other proteins) was carried out, which shows that trypsin (KR) is the best single "cutter" with coverage of 69.9% – slightly better than chymotrypsin (FYW) with 67.5% (data not shown). Combinations of cleavage reagents can further improve the sequence coverage: FYWE and KRMW are the best combinations of cleavage sites in this mass window and result in coverage of 78.7% and 77.8%, respectively. It is possible to obtain a better sequence coverage using all cutters, if the mass window is extended up to 5000 Da. In this case, the cleavage sites FYWK give the best result with coverage of 82.5%. If only proteins are considered that are not predicted to be membrane integral (e.g. cytosolic), the best single cutter for proteins is trypsin (KR) with a sequence coverage of 74.5% in the 600–4000 Da window. Only a minor increase of sequence coverage (78.5%) is possible for non membrane integral proteins if the best combination of cleavage agents (FYWE) is used.

**Figure 3:**
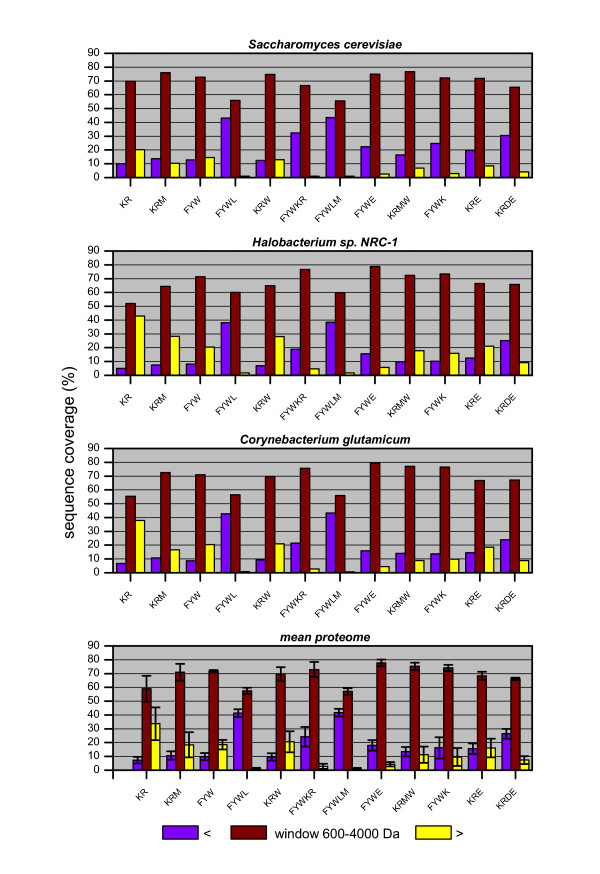
**Sequence coverage of the membrane proteome**. Sequence coverages were calculated for different cleavage conditions in the mass window of 600–4000 Da. A hypothetical mean membrane proteome was calculated from the data for all three organisms. Error bars indicate the standard deviation.

It is known that amino acid frequencies are different in integral membrane proteins and non membrane proteins. Since these differences and other protein properties should have an effect on optimal digestion conditions, the membrane proteins were analyzed separately for three organisms which represent archaea, prokaryotes and eukaryotes. (Fig. 3). For *C. glutamicum *the sequence coverage by trypsin is relatively poor with 55.4% in the 600–4000 Da mass window. Chymotrypsin (FYW) clearly outperforms trypsin and yields coverage of 70.9%. For the cutting sites in this analysis, the introduction of additional cleavage sites is only beneficial if cleavage occurs at hydrophilic and hydrophobic amino acids. The other two cases FYWL and KRE lead to a decrease in coverage in respect to KR and FYW. The best condition is a cleavage with chymotrypsin and staphylococcal peptidase I (FYWE) which results in a coverage of 79.5%.

The *Halobacterium sp*. membrane proteome is covered poorly in the 600–4000 Da mass range if trypsin is used as protease (52.0% sequence coverage), while much better results are obtained for cleavages at FYW (71.3%) with chymotrypsin. Yet, if perfect cleavage occurs at FYWL, the sequence coverage drops again to a value of 60.1%. The reason is that additional cleavages at leucine produces peptides too small to be detected – the same effect can be observed for *C. glutamicum *and *S. cerevisiae*. Higher sequence coverage was obtained with combinations of "cutters"; cleavages at KRE, KRDE, and KRM already outperform trypsin but not chymotrypsin, while KRMW, FYWE, and FYWK permit even higher sequence coverage than chymotrypsin. The cleavage conditions KRE and KRDE achieve almost the same coverage, yet the most losses were found in the high mass range for KRE, while they were in the low mass range for KRDE with more cleavage sites. The best combination is a combination of chymotrypsin and staphylococcal peptidase I (FYWE) with 78.7% coverage. As for *C. glutamicum*, the best results are obtained if cleavage at hydrophilic and hydrophobic residues occurs.

The *S. cerevisiae *membrane proteome is covered well if only tryspin is used, i.e. 69.7% coverage is obtained. Cleavages at FYW permit a marginally better coverage of 72.7%. Combination of cleavage reagents were found that yield higher coverage than trypsin or chymotrypsin alone, but the increase in coverage is not as high as for *C. glutamicum *and *Halobacterium*. The best cleavage in the 600–4000 Da mass window would be with chymotrypsin and staphylococcal peptidase I at FYWE (78.7% coverage). KRM is also a good alternative for MALDI using yeast, since the coverage of 76.0% is not much lower.

In practice, one would prefer conditions that require the least experimental effort and are most reproducible. This could be the combination trypsin / chymotrypsin (FYWKR) since the digestion can be carried out simultaneously with both proteases and a good coverage of 75.7% can be attained.

From the results for all the organisms a hypothetical mean membrane proteome was calculated to examine whether one cleavage condition exists that is significantly better suited for membrane proteins than trypsin. Indeed, cleavages at KRMW, FYWK and FYWE yield a significantly better coverage. Furthermore it can be stated for all three organisms that if the number of cleavable amino acids with the same physicochemical properties (hydrophilic or hydrophobic) is increased, the coverage does not significantly increase and indeed, sometimes decreases. This observation is based on the fact that the amino acid sequences of proteins are not random; instead they reflect evolutional processes that lead to a better adaptation of function and stability. If it were random, sequence coverage of membrane proteins from *C. glutamicum *should be almost the same for the combinations KRM and KRE, since under this assumption the average peptide length would be 9.8 amino acids for KRM and 8.8 amino acids for KRE.

### Fraction of unique peptides in the mass window

**Figure 4:**
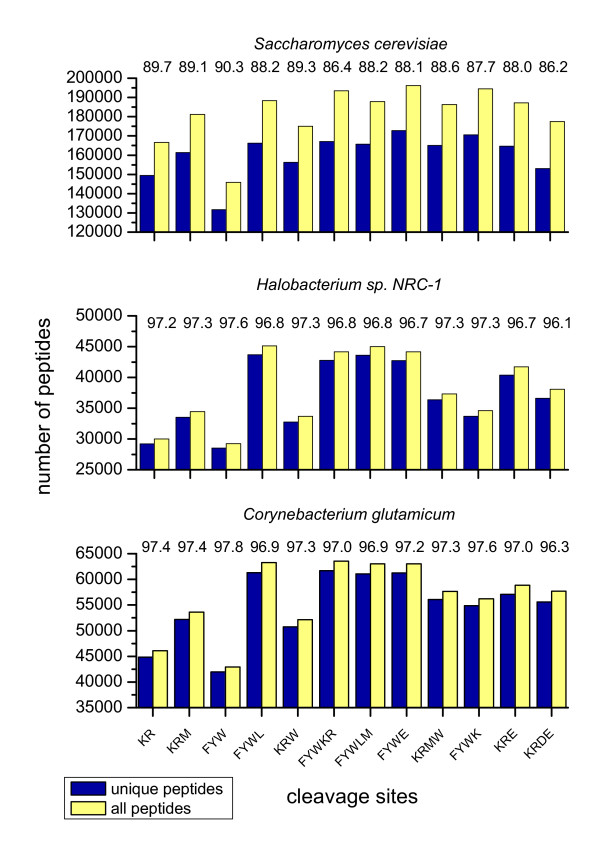
**Fraction of unique peptides in the ESI mass window**. The number of unique peptides is depicted in the ESI-ion trap mass window (500–6000 Da) for the fraction of unique peptides / total peptides in percent. For the calculations it was assumed that it is impossible to discriminate between leucine and isoleucine in MS/MS experiments.

In classical MALDI-TOF, the PMF technique is used for protein identification, whereas ESI offers in addition the possibility to relatively easily obtain the peptide sequence and thus to identify a protein solely based on one peptide fragment. A prerequisite for this approach is a unique sequence of the fragment, i.e. it must be absent in other proteins from the same organism. The goal of the following study was to verify how often protein identification is possible, if one peptide can be sequenced, and whether differences exist between the various cleavage conditions. The same cleavage conditions as for the sequence coverage studies were chosen to ease comparison. In practice, it is nearly impossible to obtain a membrane fraction which is not contaminated with cytosolic and peripheral membrane proteins. In order to better reflect this situation, the number of unique peptides was calculated for the complete proteome (instead of only the membrane proteins) of *S. cerevisiae, Halobacterium sp*., and *C. glutamicum *and is displayed in Fig. 4.

For *C. glutamicum*, the variation in the total number of peptides and of unique peptides in the assumed 600–4000 Da mass window is not very high. Here, the range for the number of peptides is from 42,919 total / 41,955 unique for cleavage at FYW to 63,563 total / 61,677 unique for cleavage at FYWKR. The ratio of unique / total peptides is almost identical for all tested cleavage conditions. The lowest number was calculated for KRDE (96.3% unique) and the highest for FYW (97.8% unique). In summary, the total number of peptides varies about 50% between the tested conditions, and about 97% of all peptides in the mass window are unique; thus, one peptide is almost always sufficient to identify the corresponding protein. Due to this, cleavage conditions can be used that achieve the best sequence coverage.

For *Halobacterium sp*., the number of peptides varies more between the different cleavage conditions than it is the case for *C. glutamicum*. The range is from 29,230 total / 28,532 unique for cleavage at FYW to 45,109 total / 43,684 unique for cleavage at FYWL. The fraction of unique peptides is almost similar for all cleavage conditions and ranges from 96.1% for KRDE to 97.6% for FYW. These calculations show that the cleavage conditions for highest sequence coverage (FYWE, FYWKR) are also suitable in respect to the number and uniqueness of the peptides.

The *S. cerevisiae *proteome consists of more proteins than *C. glutamicum *and *Halobacterium sp*. While the number of proteins in the yeast proteome is about 50% higher than in *C. glutamicum*, the number of peptides in the ESI mass window is more than doubled for the tested cleavage conditions. The range extends from 145,900 total / 131,707 unique for cleavage at FYW to 194,537 total / 170,538 unique for cleavage at FYWK. Among the three organisms, the percentage of unique peptides is lowest for yeast and rises from 86.2% for KRDE to 90.3% for FYW. In the case of yeast, cleavage at KR with trypsin already was suitable to obtain high sequence coverage, and only a slight improvement was obtained with other conditions, e.g. KRM. Both cleavages yield about the same percentage of unique peptides, which is 89.7% for KR and 89.1% for KRM.

### Dependence of the peptide number from protein size and cleavage condition

**Figure 5:**
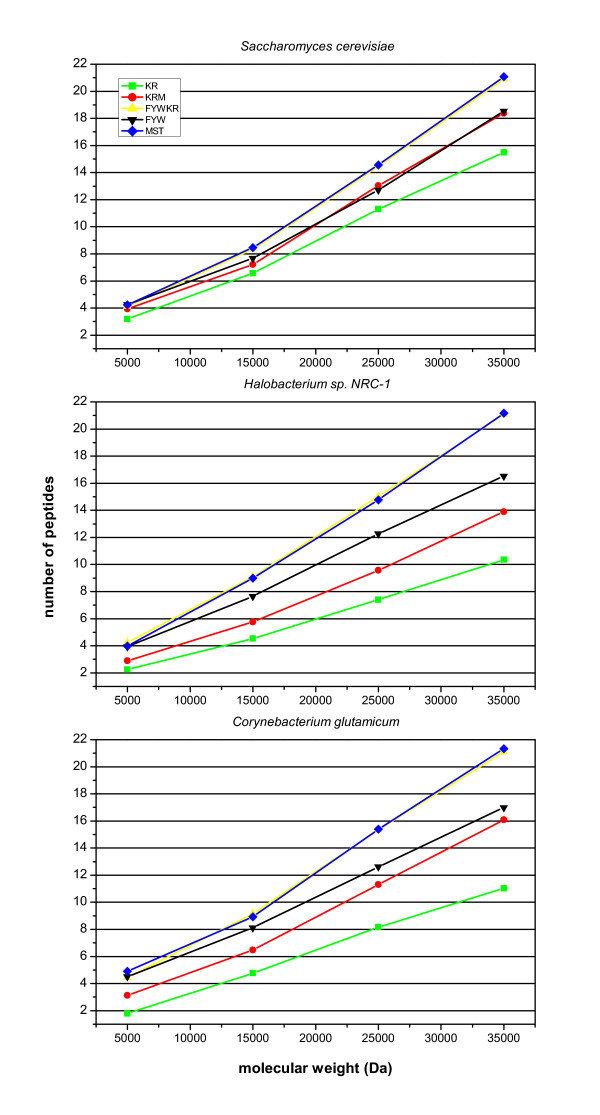
**Peptide number under different cleavage conditions**. The dependence of the mean number of obtained peptides on membrane protein size and cleavage conditions was calculated in the MALDI-TOF mass window (600–4000 Da).

For the identification of proteins by MALDI-TOF PMF, which is often used for high-throughput proteomics, about 4 peptides per protein are sufficient [12]. Even under ideal conditions a protein has to be around 4 kDa in size. In practice, proteins smaller than 20 kDa are often impossible to identify by MALDI-TOF PMF with sufficient confidence. A theoretical number of 8 peptides per protein should be considered as the lower limit, since in practice not all peptides are ionised by the MALDI process – from our experience only about half of the possible peptides can be detected in the mass spectrum. The mean number of peptides resulting from different cleavage conditions was calculated for the membrane proteins from *C. glutamicum, S. cerevisiae*, and *Halobacterium sp*. in the suitable mass window for MALDI-TOF (Fig. 5). It is evident for *C. glutamicum *that the protease trypsin (KR) is very problematic for the identification of small membrane proteins. A number of 11 membrane proteins do not possess any trypsin cleavage site and thus are not amenable for identification by MALDI-TOF PMF. The cutting sites KRM and FYW outperform KR. Cleavages at FYWKR and MST yield the highest number of peptides, but even under these conditions identification of small proteins is a problem. A number of 8 peptides are derived from KR cleavage at 25 kDa, while the cleavages at FYWKR and MST already yield this result at ~12 kDa. This distinctly lowers the detection barrier for MALDI-TOF PMF. For the practical application it is interesting to see that by consecutive chemical cleavage with CNBr followed by S-ethyltrifluorothioacetate vapour (e.g. MST) [14], acceptable cleavage of membrane proteins also occurs. This cleavage condition can be an alternative method for those membrane proteins which still reside in the lipid bilayer or are difficult to unfold, and therefore, may not be completely accessible for proteases like trypsin or chymotrypsin.

For *Halobacterium sp*., the cleavage at KR is most unfavorable among all three organisms. On average, 8 peptides are obtained for membrane proteins larger than ~27 kDa. The relative suitability of all tested cleavage conditions is similar to *C. glutamicum*. MST and FYWKR are the best cleavage conditions and produce on average 8 peptides for proteins above ~13 kDa. Under these conditions, about twice as much peptides are obtained in the MALDI-TOF mass range (600–4000 Da) in comparison to trypsin, which should greatly increase the chance of identifying integral membrane proteins from this organism by the PMF technique.

For *C. cerevisiae*, the number of produced peptides does not vary much between the different cleavage conditions in contrast to the other two organisms. Proteolysis at KR with trypsin already produces 8 peptides per protein at ~17 kDa. Once again, MST and FYWKR are most suitable to increase the peptide number; both yield 8 peptides at ~14 kDa. Interestingly, the cleavages at MST and FYWKR give almost the same results in all three organisms. Furthermore, in respect to KR, KRM and FYW yield more peptides per protein in yeast compared to *Halobacterium sp*. and *C. glutamcium*. FYW is a largely complementary cleavage condition to KR, which was found by calculating for each membrane protein the number of obtained peptides in the MALDI range (data not shown). The number of peptides differed largest between these two conditions – up to 19 more peptides could be generated for some proteins below 40 kDa by FYW cleavage, yet up to ten less peptides were observed, too.

### Bacteriorhodopsin – a practical approach

**Figure 6:**
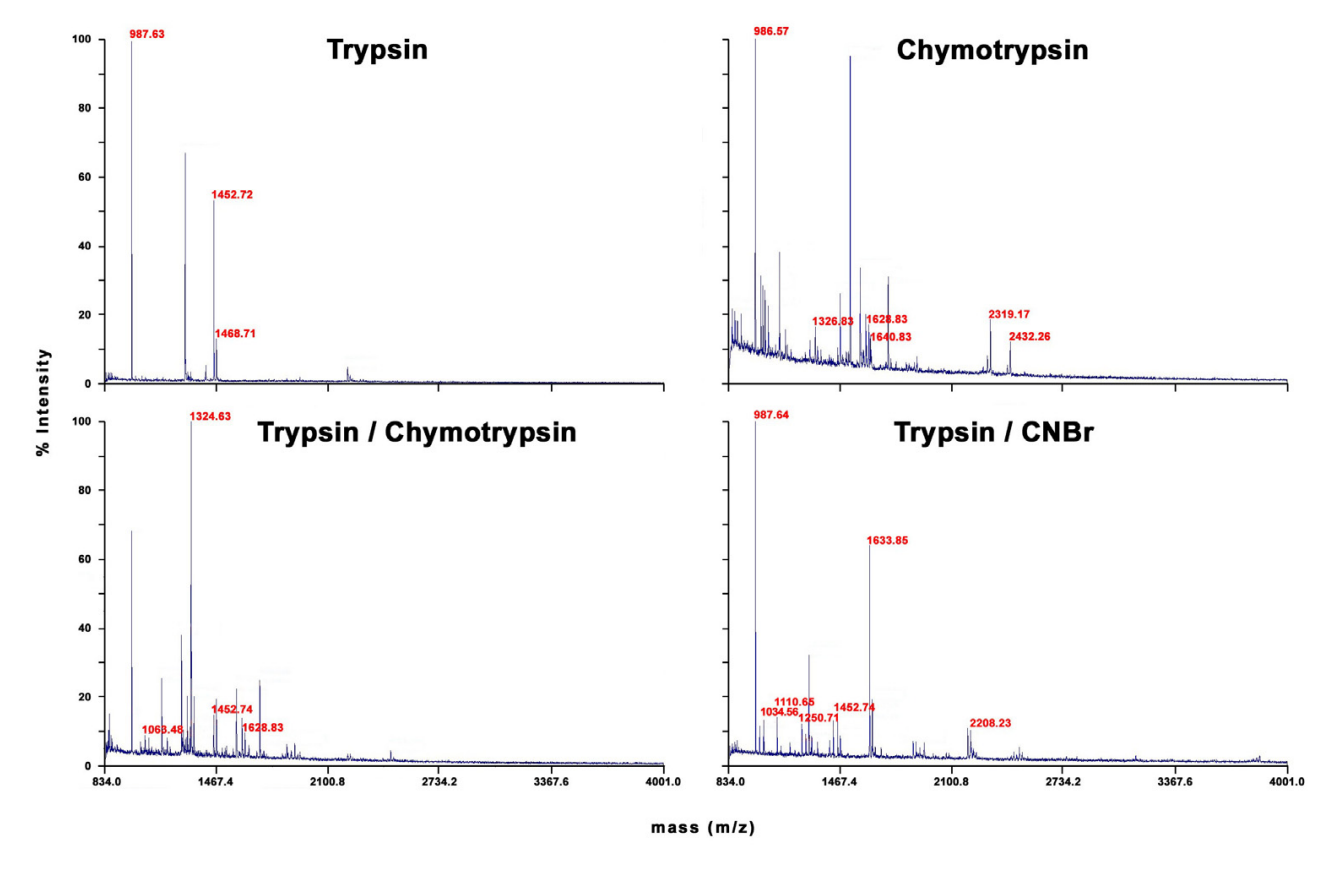
**MALDI-TOF spectra of peptides after bacteriorhodopsin cleavage**. MALDI-TOF mass spectra of bacteriorhodopsin after four different cleavage procedures: trypsin, chymotrypsin, trypsin / chymotrypsin and trypsin / CNBr. Peptide masses are given if they comply with the *in silico *prediction.

To test if *in silico *analysis of different digestion conditions is useful for the practical identification of an integral membrane protein, bacteriorhodopsin (b_R_) was used as a model. Four different digestion conditions were tested: trypsin (KR), chymotrypsin (FYWL), trypsin / chymotrypsin (FYWLKR) and trypsin / CNBr (KRM). *In silico*, the number of proteolytically derived peptides in the mass range from 800 to 4000 *m/z *were as follows: trypsin (6 peptides); trypsin / chymotrypsin (9 peptides); chymotrypsin (11 peptides) and trypsin / CNBr (13 peptides). For that reason it could be expected that the digestion with trypsin / CNBr should be optimal. The MALDI-TOF-MS spectra of bacteriorhodopsin for the four different digestion conditions are shown in Fig. 6. In all cases only these peaks which could be assigned to bacteriorhodopsin peptides were labeled. In the tryptic digest only four clear peaks were visible, while three could be related to peptides of b_R_, which is a peak match rate of 75% (Tab. 2).

**Table 2:** Result summary for the bacteriorhodopsin MALDI experiment

**Protease**	**Mascot scores (MOWSE scoring)**	**Sequence coverage [%]**	**No. of identified peptides**	**Matched peaks [%]**	**Identification rank (Mascot list)**
trypsin	34	8	3	75	2
chymotrypsin	70	27	6	35	1
trypsin/chymotrypsin	39	20	4	21	1
trypsin/cyanogen bromide	92	27	7	50	1

The other spectra showed a much higher number of peaks; especially the use of chymotrypsin leads to much more "noise" in the resulting spectra. In these cases the peak match rate decreases to 35% (6 of 17 peaks) for the single chymotrypsin digest and even to 26% (4 of 19 peaks) for the trypsin / chymotrypsin digest (Tab. 2). 50% (7 of 14 peaks) of all peaks could be related to bacteriorhodopsin peptides in the trypsin / CNBr digested sample. The trypsin digested sample showed the clearest MALDI-TOF spectrum with a total protein coverage of 8%. Nevertheless, this coverage is quite low when compared to the trypsin / chymotrypsin digest (20%) or to the chymotrypsin and trypsin / CNBr digest (27%). All the described data had an influence on the probability based scoring system i.e. the MOWSE score system Pappin, 1993 #14]. We noted that when searching the archaea database, Mascot scores above 61 were significant to identify a protein. Only cleavage with chymotrypsin (Mascot score 70) or trypsin / CNBr (Mascot score 92) resulted in a significant Mascot score for bacteriorhodopsin (see Tab. 2).

## Discussion

### Predictions and limitations of the digest model

In this study *in silico *digestion was used to find cleavage conditions for an improved analysis of integral membrane proteins for the three model organisms *S. cerevisiae, Halobacterium sp. NRC-1*, and *Corynebacterium glutamicum*. The TMHMM algorithm [15] was used to identify integral membrane proteins from these organisms, since no complete experimentally determined dataset exists. While one has to be aware that certain integral membrane proteins (e.g. single transmembrane proteins and integral porins) are missed in the bioinformatics prediction, this and other state of the art algorithms are overall very specific and sensitive [16]. To ascertain whether this *in silico *approach is pragmatic, we verified our predictions experimentally using the model membrane protein bacteriorhodopsin. We considered this experimental verification necessary, because the *in silico *model contains several limitations. For instance the used cleavage model assumes that with the exception of blocking, no missed cleavages occur. This is unrealistic since often in MALDI analysis, even with a peptide occurring at high frequency, one missed cleavage site is observed. This is mainly caused by inaccessibility of the cleavage site for the protease due to incomplete unfolding. In practice unfolding is improved by the use of chaotropes like urea [17], methanol [11], or an acid-labile surfactant [18]. Furthermore, KK and RR are often not digested completely, since trypsin is an endoprotease and has weak exoprotease activity. Others have approached these problems by calculating best case (no missed cleavage) and worst case scenarios (one or two misses) for comparison [19,13]. Different values exist in the literature for the minimum number of peptides that are necessary to identify a protein with MALDI-TOF PMF. They range from 2.2 and 2.3 [12] to 5 [20]. Further on, this number depends on the peptide mass, since fewer proteins match to large peptides [19]. As often small membrane proteins do not yield enough peptides for identification, our *in silico *study recommends alternative cleavage procedures, e.g. a combination of trypsin and chymotrypsin for all three organisms, *S. cerevisiae, Halobacterium sp*., and *C. glutamicum*. For the identification of membrane proteins larger than 20 kDa by MALDI-TOF PMF trypsin alone appears sufficient.

When the mean number of peptides per membrane protein generated by different cleaving agents was calculated, trypsin was clearly inferior to a combination of trypsin/CNBr, or chymotrypsin. An inspection of the peptide sequences from membrane protein cleavages above 4000 Da revealed that indeed a high number of these are either uncleaved transmembrane segments, if trypsin (KR) is used as a protease, or long hydrophilic stretches of soluble protein domains, if chymotrypsin (FYW) is used. Therefore all three organisms exhibit the common characteristic that the best combination of "cutters" for integral membrane proteins is a cleavage at both hydrophilic and hydrophobic amino acids. Among these combinations, one was superior to trypsin for all three organisms, the combination of chymotrypsin and staphylococcal peptidase I (FYWE). In Shotgun proteomics, the peptide mass and sequence is generally the only information to identify the original protein. The analysis of the *C. glutamicum *proteome showed that in the 600–4000 Da mass range more than 96% of the peptides contain unique sequences and thus almost always lead to protein identification. This agrees with an *in silico *analysis of *E. coli*, *S. pombe*, and *S. cerevisiae *proteome – around 1%, 1.5%, and 2% of the respective ensemble of peptides with at least seven residues (i.e. ~800 Da), were redundant [13]. However, it can be seen from the analysis for *C. glutamicum*, that also in the mass range below 600 Da at least more than 60% of the peptides are unique for trypsin cleavage, which leaves this range still attractive for analysis with instruments that allow low mass detection (e.g. Q-TOF).

### Improvements in membrane protein analysis – theory and practice

We believe the best criterion to evaluate the suitability of the predictions would be to carry out a practical test using a MALDI-TOF instrument. For this purpose bacteriorhodopsin was digested with trypsin (KR), chymotrypsin (FYWL), trypsin / chymotrypsin (FYWLKR) and trypsin / CNBr (KRM). From this experiment, it can be concluded that the predictions agree well with the practical results. As predicted, the highest sequence coverage was obtained for digestion with chymotrypsin or trypsin / CNBr (27%). Additionally, the trypsin / CNBr digest yielded the most theoretical peptides and also for this sample, the highest number of peptides could be matched. It was observed that fewer peaks could be matched in the chymotryptic digests than in the tryptic digests. This is a consequence of the lower specificity of chymotrypsin and a higher autoproteolysis rate in comparison to trypsin. Thus the predictive power of the *in silico *digests is better for enzymes that are highly specific. Nonetheless, the predictions for chymotrypsin were good enough to point out the applicability of this protease for membrane protein identification. Comparison between the bacteriorhodopsin sequence coverage of chymotrypsin and trypsin /CNBr further revealed that although under both conditions equal values are obtained, the matched sequence parts are largely different. Two different digestions, one with chymotrypsin and one with trypsin / CNBr appear attractive if high sequence coverage is desired.

So far no *in silico *study has been published that analyses cytosolic and membrane proteins separately on the complete proteome level. It became evident that it is necessary to use different cleavage conditions for membrane and soluble proteins if high sequence coverage is desired. To better simulate the experimental conditions, it was chosen to limit the considered peptides for the *in silico *analyses to mass windows that are typical for the used mass spectrometry technology. Nevertheless, in practice the window can be even smaller. For instance, peptide analysis by MALDI-TOF is often unsuccessful for masses less than 780 Da, since the matrix produces a high number of peaks in this range, too.

We set the mass range to 600–4000 Da for the analysis of membrane proteome coverage and the number of unique peptides. This in our opinion, a reasonable range for MALDI and ESI, but we are aware that in reality the size of the useful mass window can vary. To allow for this variability, the membrane proteome coverage in relation to the peptide mass was depicted. It can be said in general that optimal cleavage conditions minimize the number of peptides and proteome coverage outside the accessible mass range. For the 600–4000 Da window, cleavage conditions superior to trypsin reduced the coverage of the membrane proteome beyond 4000 Da peptide size, while not too many small peptides were generated which either escape detection or are not informative for protein identification. It has been observed in practice that the peptide sequence affects its ionisation in MALDI and ESI. Although it was not considered in our study, trypsin usually delivers peptides that tend to ionise well, and this can be used to calculate the confidence for an identified peptide [21]. While for the *in silico *analysis, a mass range of 600–4000 Da was assumed, doubly charged peptides with less than 800 Da are rarely used for database searching in ESI-MS/MS, since the two terminal amino acids fragment together, leaving only a very short stretch of peptide for sequence deduction. For the small proteome of *C. glutamicum *a sequence fragment of 4 amino acids is often sufficient to identify a protein, but this is not the case for organisms with a higher number of proteins.

The reason for the high number of unique peptides for the three organisms in the 600–4000 Da mass range is their relatively small number of coding genes. In reality, the number of peptides will be larger, due to effects like protein splicing, posttranslational modifications, missed cleavages, etc. For eukaryotic organisms it can indeed be important to verify if protein identification by a peptide eliminates the possibility of existing splice variants as demonstrated by Kuster et al. [22]. In addition, it is estimated that by using MudPIT, about 23,000 peptides can be separated in one experiment [5]; this number is smaller than the number of predicted peptides from *in silico *digestion that fall into the defined mass range. These observations raise the question, whether the proposed strategy from *in silico*, i.e. multiple cleavage combinations, is applicable in reality. Our own findings for the membrane fraction of *C. glutamicum *[23] proof that the combination of trypsin and chymotrypsin or trypsin and cyanogen bromide [24] can be superior to trypsin alone. Others have found even for higher eukaryotes (brain homogenisate) that cleavage with unspecific proteinase K was extremely useful for membrane protein identification. However, it must be stressed that with current instrument limitations, prefractionation steps for membrane enrichment are mandatory.

## Conclusion

By carrying out *in silico *proteome analysis, integral membrane protein cleavage conditions were identified that are superior to trypsin for all three examined organisms, *Halobacterium sp. NRC-1, Saccharomyces cerevisiae*, and *Corynebacterium glutamicum*. While the best result was obtained with a combination of chymotrypsin and staphylococcal peptidase I, in general all superior conditions are combined cleavages at hydrophobic and hydrophilic amino acids. The combined cleavages improved the sequence coverage for ESI-MS/MS and the identification of small integral membrane proteins by MALDI-TOF PMF. The practicability of cleavage conditions was assessed by digestion of bacteriorhodopsin and MALDI-TOF PMF. It can be concluded that the *in silico *predictions agree well with the practical results, particularly if highly specific proteases are used. Since some of the improved cleavage conditions (e.g. chymytropsin + trypsin) are not more elaborate than the classical trypsin digestion, they should useful alternatives to trypsin for a better analysis of integral membrane proteins.

## Methods

### Datasets and bioinformatics

A file containing the translated predicted ORFs for *Halobacterium sp. NRC-1 *(NC_002607) was downloaded from the NCBI FTP website. Predicted protein sequences for *Saccharomyces cerevisae *were obtained from the European Bioinformatics Institute (EBI) website [25]. The translated predicted ORFs for *Corynebacterium glutamicum *were supplied by J. Kalinowski (Bielefeld University) and are part of the publicly available dataset at EBI. The *in silico *digestion software was written in PERL and is taking advantage of BioPerl 1.4 modules. Cleavage rules (simple model for trypsin and chymotrypsin) for the *in silico *digestions were employed according to the Expasy PeptideCutter website [26], aside from these rules perfect cleavage was assumed. Membrane proteins were predicted for all three organisms using the software TMHMM 2.0 [15].

### Experimental validation of cleavage conditions

#### SDS-PAGE

In each case 20 μg (protein amount) of isolated purple membranes were dissolved in sample buffer (10% v/v glycerol, 5% v/v mercaptoethanol, 3% w/v SDS, 62 mM Tris-HCl, pH 6.8, 0.01% bromophenol blue) and incubated for 30 min at 60°C. SDS-PAGE was performed according to Laemmli [27]. After electrophoresis the proteins were stained by colloidal Coomassie blue using the protocol of Neuhoff [28].

#### In-gel digestion of bacteriorhodopsin

The Coomassie stained protein bands were excised from the SDS-polyacrylamide-gel and completely destained with 100 μL 25 mM ammonium hydrogencarbonate and 50% v/v acetonitrile (three times for 20 min by 37°C). The gel pieces were dried in a Speed Vac and subsequently covered with a 25 mM ammonium hydrogencarbonate buffer containing either 12.5 μg trypsin (three samples) or 12.5 μg chymotrypsin (one sample). Proteolysis was carried out overnight at 37°C. The next day, peptide samples of one tryptic digest and of the chymotryptic digest were eluted by adding 10 μL elution buffer (50% v/v acetonitrile, 0.5% TFA) with subsequent sonication for 20 min. The gel pieces of the second and third tryptic digests were completely dried in a vacuum concentrator and used for a second cleaving procedure. The dried gel piece of the second tryptic digest was covered with chymotrypsin in ammonium hydrogencarbonate buffer and incubated for 7 h at 37°C. Elution of the generated peptides was carried out as described above. With the third sample a CNBr cleavage was carried out in the dark at room temperature for 4 h, while a small crystal of CNBr was dissolved in 70 % TFA and added to the dried gel piece, followed by several washing steps according to the protocol of Van Monfort [8].

#### MALDI-TOF-MS and protein identification

Aliquotes of 0.6 μL peptide digest were applied onto a target plate and immediately mixed with an equal volume of α-cyano-hydroxycinnamic acid (10 mg/ml in 60% v/v acetonitrile and 1% v/v TFA). MALDI-TOF mass spectra were recorded using an Applied Biosystems Voyager DE-Pro instrument. Each spectrum was first externally calibrated by an Applied Biosystems external peptide standard and in a second step internally calibrated using trypsin autoproteolytic products. Monoisotopic peptide masses were used to search the ncbi database using the Mascot algorithm (version 2.1) [29]. Peptides with missing cleavage sides of 2 were accepted and the mass tolerance of the peptide mass ions was set to < 100 ppm.

## Competing interests

The author(s) declare that they have no competing interests.

## Authors' contributions

FF has carried out the bacteriorhodopsin digestion, MS analysis, and interpretation of the results. AP has performed the *in silico *proteome analysis.
